# Impacts of Rearing Enrichments on Pullets’ and Free-Range Hens’ Positive Behaviors across the Flock Cycle

**DOI:** 10.3390/ani12030280

**Published:** 2022-01-23

**Authors:** Dana L. M. Campbell, Sue Belson, Tim R. Dyall, Jim M. Lea, Caroline Lee

**Affiliations:** Agriculture and Food, Commonwealth Scientific and Industrial Research Organisation (CSIRO), Armidale, NSW 2350, Australia; sue.belson@csiro.au (S.B.); tim.dyall@csiro.au (T.R.D.); jim.lea@csiro.au (J.M.L.); caroline.lee@csiro.au (C.L.)

**Keywords:** dust bathing, foraging, play, laying hen, novel objects, perching structures, navigation

## Abstract

**Simple Summary:**

Enrichment during the indoor rearing of young laying hens (pullets) destined for free-range systems may improve pullet development and increase motivated natural behaviors (termed ‘positive behaviors’) such as foraging, dust bathing and chick play. Hy-Line Brown® chicks (*n* = 1700) were floor-reared indoors across 16 weeks with three enrichment treatments (*n* = 3 pens/treatment): (1) standard control, (2) weekly novel objects—‘novelty’, (3) perching/navigation structures—‘structural’. Pullets (16 weeks old: *n* = 1386) were then transferred to nine identical pens within rearing treatments, with outdoor range access from 25 to 65 weeks. Video cameras recorded the pullet pens, adult indoor pens, and outside range. During rearing, observations of play behavior in chicks at 2, 4 and 6 weeks showed no overall effect of rearing treatment. At 11 and 14 weeks only the novelty hens were observed to increase their foraging across age with no differences between treatments in dust bathing. Observations of adult hens at 26, 31, 41, 50, 60 and 64 weeks showed that the structural hens exhibited more dust bathing and foraging overall than the control hens, but that both novelty and/or structural hens showed small increases relative to control hens depending on the behavior and location. Across age, adult hens differed in the degree of dust bathing performed inside or outside and foraging outside but not inside. For litter-reared pullets, additional enrichments may result in some long-term increases in positive behaviors.

**Abstract:**

Enrichment during the indoor rearing of pullets destined for free-range systems may optimize pullet development including increasing motivated natural behaviors (termed ‘positive behaviors’) including foraging, dust bathing and chick play. Hy-Line Brown® chicks (*n* = 1700) were floor-reared indoors across 16 weeks with three enrichment treatments (*n* = 3 pens/treatment): (1) standard control, (2) weekly novel objects—‘novelty’, (3) perching/navigation structures—‘structural’. At 16 weeks, pullets (*n* = 1386) were transferred to nine identical pens within rearing treatments with outdoor range access from 25 to 65 weeks. Video cameras recorded the pullet pens, adult indoor pens, and outside range. During rearing, observations of play behavior (running, frolicking, wing-flapping, sparring) in chicks at 2, 4 and 6 weeks (total of 432 thirty-second scans: 16 observations × 3 days × 9 pens) showed no overall effect of rearing treatment (*p* = 0.16). At 11 and 14 weeks only the ‘novelty’ hens were observed to increase their foraging across age (*p* = 0.009; dust bathing: *p* = 0.40) (total of 612 thirty-second scans per behavior: 17 observations × 2 days × 2 age points × 9 pens). Observations of adult hens at 26, 31, 41, 50, 60 and 64 weeks showed that the structural hens exhibited overall more dust bathing and foraging than the control hens (both *p* < 0.04) but both novelty and/or structural hens showed small increases depending on the behavior and location (total of 4104 scans per behavior: 17 observations × 2 days × 6 age points × 9 pens × 2 locations = 3672 + an additional 432 observations following daylight saving). Across age, adult hens differed in the degree of dust bathing performed inside or outside (both *p* ≤ 0.001) and foraging outside (*p* < 0.001) but not inside (*p* = 0.15). For litter-reared pullets, additional enrichments may result in some long-term increases in positive behaviors.

## 1. Introduction

Free-range laying hen production systems are prevalent within Australia due to their popularity with consumers [1]. Across Australia and internationally, free-range hens are perceived to have improved welfare and the eggs are preferred by consumers for perceived better quality and health benefits [2,3]. However, the welfare status of hens in free-range systems can be complex as there are both benefits and challenges to providing birds with outdoor access [4]. Free-range systems can provide hens with increased space, a more natural outdoor environment and greater ranging can improve plumage quality [5,6,7]. Conversely, the outdoor environment is more unpredictable than the controlled indoor setting and may place greater physical stressors on hens which could increase mortality [8,9]. 

One of the potential benefits of free-range systems is the increased space and outdoor environment that provides more freedom to exhibit behaviors that are viewed as part of a positive behavioral repertoire for hens [10]. These positive species-specific behaviors are typical natural behaviors that are not abnormal or negative (e.g., severe feather pecking, smothering) and hens show motivation to perform [11]. These include dust bathing and foraging (scratching and pecking at the ground) where environments that facilitate these (and other) natural behaviors are believed to provide positive welfare experiences for commercial hens [10]. These behaviors are thwarted in conventional cage systems but are facilitated in indoor litter-based systems and may be greater in systems with outdoor access. Previous observations in an experimental free-range setting showed behavioral repertoires differed between hens located inside the shed versus out on the range with hens located outside showing greater foraging and dust bathing relative to what was exhibited by hens inside on the litter [12]. On a commercial free-range farm, hens were primarily observed to be foraging when outside on the range [13], with foraging likely to occur more frequently than dust bathing [14], although behaviors can vary depending on the vegetation and topography outside [15].

While behaviors of dust bathing and foraging are innate and are performed even in the absence of suitable substrates [16,17], appropriate behavioral development can be affected by the environment pullets are reared in. The rearing environment overall is critical for physical, physiological, and behavioral development of the pullets (young, developing laying hens). There can be long-term effects on bird welfare if rearing environments are sub-optimal, or not best matched for the laying environment the pullets are transferring to later in life [18,19]. For example, rearing with access to ramps can improve use of the elevated areas for hens housed in aviaries and decrease keel bone damage [20]. Access to litter in the first four weeks of life can have long-term impacts on the development of feather pecking behaviors [21,22], which may be related to litter stimulating natural foraging behavior versus undesirable pecking of conspecifics. Adult hens may readily utilize an available dust bathing substrate even if they were reared without substrate exposure [23,24]; however, early experience without a suitable substrate may explain why some hens still sham dust bathe in the presence of litter [25]. Evidence to date demonstrates specific types of environmental enrichment can have effects on specific related behaviors as birds mature (e.g., older pullets used ramps to a greater extent when they had access to them as chicks; [26]) but can also have more generalized effects, such as rearing complexity reducing fear responses in young adult hens [27]. More generalized effects of optimizing bird development could include impacts on positive species-specific behaviors such as dust bathing and foraging. While the presence of litter can affect these, additional enrichments to a litter substrate may have even greater impacts. Peat and hay enrichments increased ground pecking in broilers, even when the birds were not in proximity of the enrichments [28]. If enrichments have generalized impacts on increasing positive behaviors the effects could be first apparent in the early weeks of life through changes in chick play behavior, which are expressed early in life and generally viewed as being positive [29,30]. 

Play behavior is performed across many animal species and may be associated with better preparing animals for the unexpected later in life [29]. Play may also be indicative of improved welfare, although not in every case, with some animals showing increased play following stressful experiences [30]. While there is limited research conducted on play behavior in chickens, spontaneous play has been observed in broiler chickens [28,31,32]. Play typically occurs in the early weeks of a chicken’s life and will decrease across age [28,31,32]. Play has previously been categorized to include behaviors such as running, worm/food running (running with an object in the beak) wing-flapping, frolicking (running with wings flapping) and sparring/play fighting, all of which are performed spontaneously in young chicks or can be stimulated in experimental contexts [28,31,32]. In the limited research available, the effects of enrichment on play are unclear. No effects have been found on spontaneous play [28,31,32] but non-enriched birds have shown more play during specific play tests [32].

Pullets destined for free-range systems in Australia (and elsewhere) are typically reared indoors before being transferred to a laying facility with outdoor access. The discrepancies between the rearing and laying housing systems may impact how the adult hens adapt to their new housing. With outdoor access for pullets being logistically difficult, enriching the rearing environment may be a strategy to optimize pullet development, improving welfare and adaptability. A rearing enrichment trial was designed to measure the impacts of different types of rearing environments on the behavior, health, production, and welfare of a flock of free-range hens in an experimental setting and showed long-term effects of the type of environment the pullets were reared in across multiple measures [5,33,34]. The aim of this study was to assess how these different rearing enrichments affected chick play behavior as well as foraging and dust bathing in the pullets and adult hens across the flock cycle. It was predicted that both types of rearing enrichments would increase these positive species-specific behaviors through generalized impacts on optimizing pullet behavioral development. While the enrichment types were distinct, there were no clear predictions on how they may differentially affect the birds, as there is limited literature available and neither of the enrichment types specifically target litter-related behaviors. 

## 2. Materials and Methods

### 2.1. Ethical Statement

All research was approved by the University of New England Animal Ethics Committee (AEC17-092).

### 2.2. Animals and Housing

#### 2.2.1. Rearing (0–16 Weeks of Age)

This study used 1700 Hy-Line^®^ Brown layers that were first reared indoors for 16 weeks in the Rob Cumming Poultry Innovation Centre of the University of New England, Armidale, Australia, before transfer to the Laureldale free-range facility of the University of New England, where they remained until the conclusion of the trial at 65 weeks of age. The housing set-up for the birds has been described previously (e.g., [5,33,34]). In November 2017, day-old, beak-trimmed chicks from a commercial hatchery were placed into nine floor-litter pens (6.2 m L × 3.2 m W) within three rooms. Chicks arrived in multiple boxes with boxes randomly allocated to treatment pens (approximately two boxes/pen). All pens within a room were visually separated with shade cloth attached to the wire pen dividers. Rice hulls covered the ground as floor litter, four round feeders per pen provided ad libitum access to commercially formulated mash and drinking water was provided via nipples (20 nipples/pen). Resources either met or exceeded the current Australian Model Code of Practice for the Welfare of Animals—Domestic Poultry [35]. Three separate rearing enrichment treatments were applied with one treatment replicate per room, balanced for location. (1) a ‘control’ group with just the floor litter, (2) a ‘novelty’ group where novel objects were changed at weekly intervals (e.g., balls, bottles, bricks, brooms, brushes, buckets, containers, pet toys, plastic pipes, strings, water bottles) and (3) a ‘structural’ group where five custom-designed H-shaped perching/navigation structures (L, W, H: all 0.60 m) with two solid panels and an open-framed side were provided in different orientations within each pen. Pullets could perch on these structures and the solid side in some orientations created a visual/physical barrier requiring the birds to navigate around it, thus the structures were designed to add complexity and stimulate both perching and navigation in the pen. There was approximately 6 cm perching space/bird during rearing provided by these structures. By 16 weeks of age, bird density was approximately 15 kg/m^2^ (average 174–190 pullets/pen resulting from both chick mortality and chick placement error). Temperature and lighting schedules followed the Hy-Line^®^ Brown alternative management guidelines [36] except the LED lighting was maintained at 100 Lux as the pullets were being reared for a free-range system. Rooms were mechanically ventilated but there was no cooling system present. Litter was visually assessed during daily routine bird health checks and was deemed dry and friable throughout the rearing period across all pens. Chicks and pullets were vaccinated as per regulatory requirements and standard recommendations.

#### 2.2.2. Free-Range Facility—Indoor Pens (16–65 Weeks of Age)

At 16 weeks of age, 1386 pullets were transferred to nine identical, visually isolated pens within a single shed at the Laureldale free-range facility of the University of New England with rearing treatments balanced for location across the shed. Additional pullets, surplus to the space restrictions of the layer shed, were rehomed including those birds that were either heavier or lighter than the mean body weight, and then some additional randomly selected pullets to reach the desired quota. Pullets were socially remixed within pen replicates of their rearing treatments (three pen replicates per rearing treatment) to simulate the social remixing that occurs commercially. However, pullets were placed into three pens containing only birds from the same rearing treatment. Bird density was approximately 9 birds/m^2^ (*n* = 154 hens/pen; 3.6 m W × 4.8 m L). Each pen contained nest boxes, perches, feeders, and water nipples to meet or exceed requirements of the Australian Model Code of Practice for the Welfare of Animals-Domestic Poultry [35]. Pen space logistics restricted perching space to 10 cm per bird, but hens also perched on the tops of the waterline and feeders. Rice hulls formed the floor litter substrate with regular raking management and one complete mid-lay litter replacement to ensure dryness and friability of the litter. By 30 weeks of age, the LED lighting schedule had gradually reached 16 hours light and 8 hours dark with an average pen intensity of 10.0 (±0.84 SE) Lux (Lutron Light Meter, LX-112850; Lutron Electronic Enterprise CO., Ltd., Taipei, Taiwan) as measured at birds’ eye height from three pen locations (front, middle, back) when the pop-holes were closed. This lux was the highest that could be achieved with the shed lighting system. The shed was fan-ventilated with no temperature or humidity control. 

#### 2.2.3. Free-Range Facility—Outdoor Range (16–65 Weeks of Age)

Each indoor pen was connected to an outdoor range area accessible via two pop-hole openings (18 cm W × 36 cm H). The nine range areas were visually isolated from each other via shade cloth on the wire fences. The pop-holes first opened at 25 weeks of age (May 2018) and provided daytime range access on an automated schedule from 09:15 until after sunset resulting in approximately 9 h of available ranging time across winter and approximately 11 h of available ranging time daylight saving time onward (October 2018). The range area comprised of a 1.2 m length concrete path, followed by 3.6 m length of river rock and then a 26.2 m length of grassed area with no trees or artificial shelters. Monthly photos of the range areas allowed visual estimation of vegetation coverage. Initially the range areas were 90% covered in grass, which was destroyed by hens or seasonal die-off after 8 weeks of range access. Six months after first range access (hen age: 48 weeks), there was some spring grass regrowth with up to 40% coverage in some pens (3 pens 0%, 4 pens 20%, 2 pens 40%) but by summer (8 months after first range access: hen age: 56 weeks) the ranges were only bare dirt with some scattered hen-resistant weeds.

### 2.3. Video Recording and Data Collection

Hikvision Network cameras (Model DS-2CD2232-I5 4 mm, Hikvision, Hangzhou, China) were installed to capture the indoor rearing pens during light hours at 2, 3, 4, 6, 8, 11 and 14 weeks of age. The same cameras were installed to capture the indoor pens and range area of each pen at the layer facility at 26, 31, 41, 50, 60 and 64 weeks of age. Across the ranging period there was typically little rain due to severe drought in the region. Due to camera angles, across all pens equally, approximately 0.5 m in front of the pop-holes inside and approximately 1.2 m in front of the pop holes outside was unavoidably excluded from video capture. Video recordings were later decoded by observers who were blind to rearing treatment or blind to the aims of the trial where rearing enrichments were visible in the video. The observers were all trained by a single researcher who simultaneously did sections of video with the trainee to ensure the correct behaviors were being identified. For instances where two observers were collecting data on the same behavior, both observers watched one identical section of video first independently (one day of observations across one pen). If inter-observer reliability was initially below 90% as assessed by correlation in Microsoft Excel (agreement values ranged from 76–89%), the two observers then discussed the section of video to reach 100% agreement in the identification of birds performing each behavior at each time point the observers previously showed discrepancies on before proceeding with their independent observation days. Where two observers watched one behavior, the allocation of pens and treatments to observe were balanced to minimize any potential observer bias per a specific rearing treatment.

The observers collected data as follows:

1. Rearing—Enrichment interactions: counts of birds using/interacting with enrichments in the rearing pens at 3, 6, 8, 11 and 14 weeks of age. These data were collected by one observer to document the use of the enrichments during rearing with no intended comparison between treatment groups. At each age point a single day of video per enriched pen (novelty and structural pens, not control pens) was observed with point counts made every 30 min from 08:00 until 17:30 (total 20 counts per day × 5 days × 6 enriched pens = total dataset of 600 counts). Days were selected to be at least 2 days after the new novel objects had been added but also at least 2 days after other/disturbances interventions such as body weight assessment as part of a separate dataset [37] and vaccinations. Interaction with the enrichments was defined as a bird perched on, pecking at, or standing/sitting directly next to an enrichment (i.e., less than a bird body width away). The structural enrichment remained the same throughout the rearing period, but the novel objects changed weekly and thus were variable across the 5 days assessed. The video was played at each time point for up to 10 s as needed to confirm bird behavior at the specified time point. 

2. Rearing—Play behavior of chicks: counts of chicks exhibiting play behavior were observed in each pen across one day at 2, 4, and 6 weeks of age. Ages were selected based on previous literature on broiler chickens [31,32] of when play behavior may be most prevalent. While the match between broiler chickens and laying hens is limited given broiler chickens reach maturity (and hence slaughter) at 6 weeks of age, this selected age period provided a starting reference point for documenting (potentially peak) play in laying hen chicks. Observations by a single observer of running, frolicking, wing-flapping and sparring were made based on the ethogram as described in Table 2 of Liu et al. (2020) [32]. Wing-flapping was included, as although it is often classified as a comfort behavior in older birds, it has been observed to be associated with play or aggressive interactions in chicks [31,32,38]. Data were collected across a 30 s period every 30 min throughout the day totalling 432 observations (16 observations per day × 3 days × 9 pens). Only a single day was chosen at each age point as it was uncertain how much play behavior would be observed (if any) and there were shorter time intervals between the observation ages relative to the dust bathing and foraging observations in the pullets and adults. 

3. Rearing—Pullet foraging and dust bathing: at 11 and 14 weeks of age, all pullet pens were observed by a single observer across two days per age point to count the number of birds dust bathing or foraging (defined as feet scratching backwards in the litter typically followed by pecking in the litter) across 30 s every 30 min from 09:30 until 17:30 (total 17 observations × 2 days × 2 age points × 9 pens = 612 observations for each behavior). This definition of foraging has been used in previous studies [12,39] although some authors include other exploratory behaviors within their foraging definition [40]. 

4. Free-range facility—Hen foraging and dust bathing: at each age, approximately one week of video was recorded with the specific days of observation within the week selected based on a full set of recordings with no missing video due to technical issues, and predominantly dry weather. Across two days each at 26, 31, 41, 50, 60 and 64 weeks of age, the number of hens dust bathing or foraging inside were counted by two observers across a 30 s period every 30 min from 09:30 (pop-holes opened at 09:15) until 17:30 (just before sunset) or until 19:30 from 50 weeks onwards following daylight saving time change (total 17 observations × 2 days × 6 age points × 9 pens = 1836 + an additional 216 observations following daylight saving: total 2052 observations each of dust bathing or foraging across the flock cycle). At each observation point, the corresponding observations of hens’ dust bathing or foraging were made outside on the range (n = 2052 observations each for dust bathing and foraging outside) by a different two observers. Selected days within age points had one full day between them that was not observed (i.e., the selected days per age week were not consecutive).

5. Free-range facility—Time budgets of hens: across two days at 50 weeks of age, a 10 m length portion of the range area for each pen was selected for time budget observations by a single observer (the same area was selected for each range, in mid-view of the video capture). This age point was selected as foraging and dust bathing on the range were observed at higher levels and daylight hours were extended for more observations. Scan sampling was applied every 30 min from 09:30 until 19:00 with hens in the designated area first counted and then a behavior allocated per hen based on the ethogram in Table 1. At each time point the video was played for a few seconds to confirm the behavior the hen was exhibiting (total 20 observations points × 9 pens × 2 days = 360 observations points).

### 2.4. Data and Statistical Analyses

All analyses were conducted in JMP 14.0.0 (SAS Institute, Cary, NC, USA) with α = 0.05. All proportions were calculated taking cumulative mortality into account. All data were checked for normality and transformed where necessary for parametric tests. The studentized residuals were visually inspected to ensure homoscedascity. Non-parametric tests were conducted where transformations could not make the data normally distributed.

The count data for interaction with enrichments were converted to proportion of birds within the pen at each time point and visually displayed. No statistical analyses were conducted on these data as there were no specific comparisons to be made among treatment groups. The counts of chicks exhibiting play behaviors (running, frolicking, wing-flapping and sparring) were converted to proportions of chicks within each pen performing each behavior at each age point. Observations across the day were summed into a daily mean per pen per age point (*n* = 27: 3 × daily means × 9 pens) and were logit transformed. A constant of 0.001 was added to the sparring proportions only prior to transformation to account for zero values. A General Linear Mixed Model (GLMM) was applied to each behavior (and all play behaviors summed together) with rearing treatment, age and their interaction as fixed effects, including pen nested within treatment as a random effect. Where significant differences were present, post-hoc Tukey’s tests were applied to the least squares means.

The counts of pullets dust bathing and foraging were converted to proportions of pullets performing the behaviors, logit transformed and mean daily values were calculated per pen for each behavior (*n* = 36: 4 daily means × 9 pens). A General Linear Mixed Model (GLMM) was applied with rearing treatment, age, and their interaction as fixed effects including pen nested within treatment and observation day as random effects. Where significant differences were present, post-hoc Tukey’s tests were applied to the least squares means.

The counts of hens dust bathing or foraging inside and outside were converted to proportions of all hens in the pen and summed across the two locations. The conversion to proportions of all hens in the pen rather than proportions of hens specifically inside or outside was to display the proportions of the total group that were exhibiting each behavior in each location (i.e., conversions based on hens present inside or outside would inflate the proportions of hens exhibiting the behavior). The original dataset (2052 observations per behavior) was summarized to include one mean value per pen per day each for dust bathing and foraging (*n* = 108 per behavior: 2 days × 6 age points × 9 pens). The proportions were logit transformed but were not normally distributed and were analyzed for an effect of rearing treatment using separate Kruskal-Wallis tests, including blocking for the effect of age (only one blocking factor was permitted in the analyses). Where significant differences were present, post-hoc tests were conducted between all pairs using the Steel-Dwass method. The proportions of hens dust bathing or foraging inside the shed or outside on the range were then analyzed separately for an effect of rearing treatment using separate Kruskal-Wallis tests that included blocking for the effect of age. A constant of 0.001 was added to these data prior to logit transformation to account for values of zero. Finally, effect of age for dust bathing or foraging in indoor and outdoor locations was analyzed using separate Kruskal-Wallis tests blocking for effects of rearing treatment. 

The counts of hens performing specific behaviors at 50 weeks of age were converted into proportions of hens in the observation area exhibiting each behavior. Due to low incidences of some behaviors, observations of body shaking, preening, sunbathing, tail shaking and wing flapping were combined into a single category of ‘comfort behaviors’. The original dataset was summarized into one mean value per behavior per pen per day (summarized dataset: 2 days × 9 pens = 18 datapoints per 11 behaviors). The behaviors of jumping/flying, piling, pecking other chickens and fighting occurred too infrequently (~ 1% of the hens’ time budget combined) and were not included in any further analyses. The proportions of comfort behaviors, dust bathing, foraging, pecking, running, standing and walking were analyzed for an effect of rearing treatment using separate non-parametric Kruskal-Wallis tests. 

## 3. Results

### 3.1. Rearing

**Figure 1:**
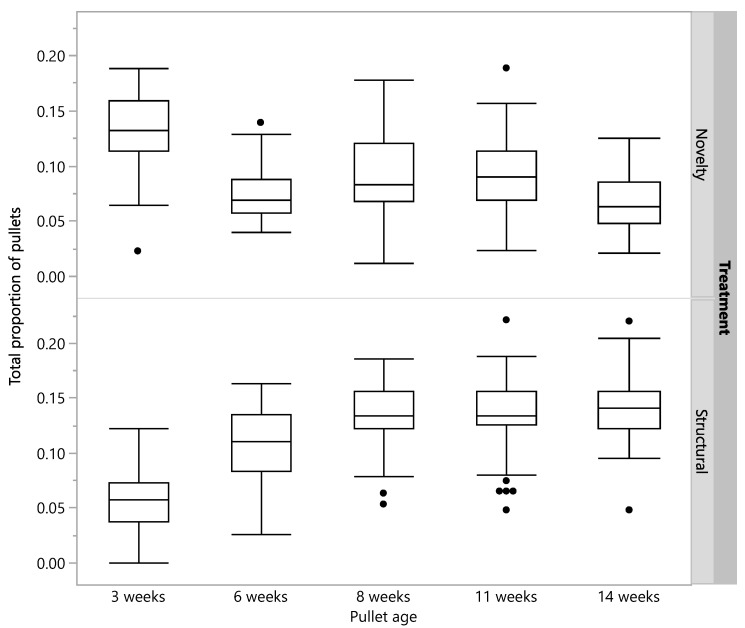
Box plots indicating the proportion of pullets in the pen that were interacting with enrichments in the novelty and structural treatment pens across five age points (3 to 14 weeks of age). The horizontal line within each box indicates the median value with the box ends representing the 1st and 3rd quartiles. The whiskers extend to the outer datapoints that fall within a distance 1.5 × outside the 1st or 3rd quartiles. If datapoints do not reach these computed ranges, the whiskers represent the upper and lower data points (excluding outliers).

Observations of the proportions of pullets utilizing enrichment in the two enriched rearing treatments showed that the birds were interacting with the provided enrichment across the rearing period with approximately 10% of pullets using them at any single point in time (overall mean ± SEM; novelty pullets: 9.22% ± 0.22; structural pullets: 11.47% ± 0.25, Figure 1).

**Figure 2:**
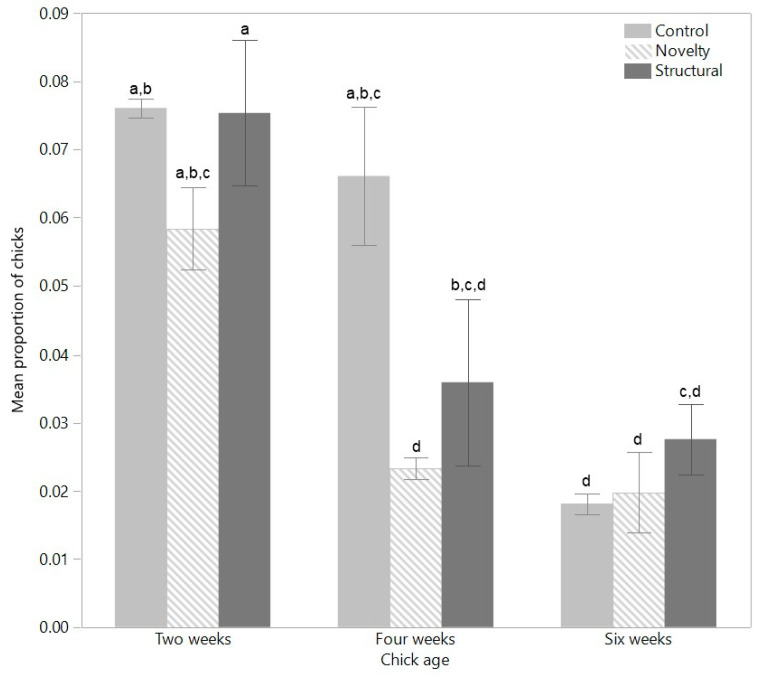
The mean (±SEM) proportion of chicks from three rearing treatments (control, novelty, structural) running within the pens across three age points (2, 4, 6 weeks). ^a–d^ Dissimilar superscript letters indicate significant differences across rearing treatments and age. Raw data are presented with analyses conducted on transformed means.

For play behaviors, there was an interaction between rearing treatment and age for the proportion of chicks running (F_(4,12)_ = 5.28, *p* = 0.02), with the enriched chicks showing less running at four weeks of age compared with the control chicks (Figure 2). There was no overall effect of rearing treatment (F_(2,6)_ = 2.57, *p* = 0.16), but running decreased linearly across age (F_(2,12)_ = 59.38, *p* < 0.0001, Figure 2). There was only an effect of age on the proportion of chicks frolicking (F_(2,12)_ = 5.76, *p* = 0.02) with less frolicking at six weeks (Table 2). There was no effect of rearing treatment (F_(2,6)_ = 2.81, *p* = 0.14), or interaction between age and rearing treatment (F_(4,12)_ = 0.44, *p* = 0.78). There was a significant effect of rearing treatment on the proportion of chicks showing wing-flapping (F_(2,6)_ = 9.50, *p* = 0.01) with the structural chicks showing less than control and novelty chicks (Table 2). There was also a significant effect of age (F_(2,12)_ = 4.67, *p* = 0.03) with less wing-flapping at two weeks of age compared with six weeks of age (Table 2). There was no interaction between rearing treatment and age (F_(4,12)_ = 0.57, *p* = 0.69). There was a significant interaction between age and rearing treatment on the proportion of chicks sparring (F_(2,12)_ = 3.29, *p* = 0.048), with the control chicks only showing more sparring at six weeks relative to two weeks of age. There was a significant effect of age (F_(2,12)_ = 5.44, *p* = 0.02) with more sparring at four weeks than at two weeks, but no overall effect of rearing treatment (F_(2,6)_ = 0.25, *p* = 0.79). When all play behaviors were combined, there was only a significant effect of age (F_(2,12)_ = 29.05, *p* < 0.0001) with play linearly decreasing across age. There was no effect of rearing treatment (F_(2,6)_ = 2.94, *p* = 0.13) and no interaction between rearing treatment and age (F_(4,12)_ = 1.22, *p* = 0.35). Running was the most frequently observed play behavior but was still observed in less than 10% of the chicks during observations, with sparring only observed in a few chicks (Table 2). 

**Figure 3:**
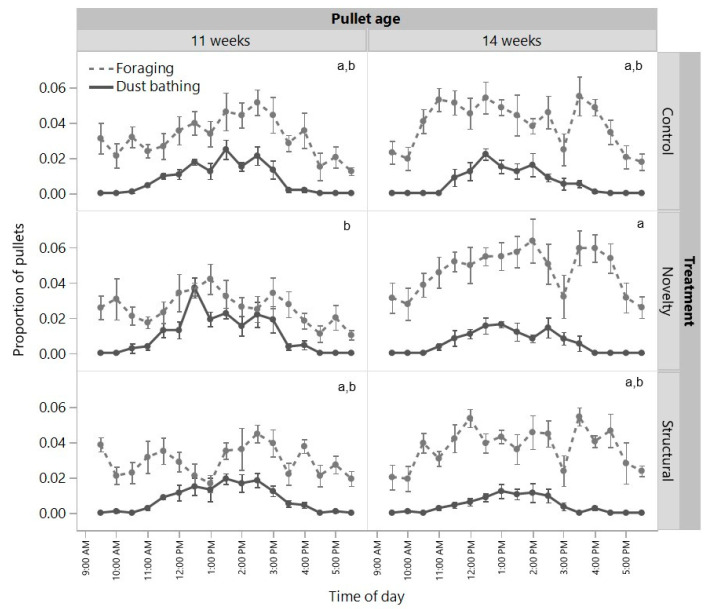
The proportion of pullets from three rearing treatments (control, novelty, structural) dust bathing or foraging across the day as assessed at 11 and 14 weeks of age. ^a,b^ Dissimilar letters indicate significant differences between treatments across age for foraging behavior. The raw mean (±SEM) values are presented across the day with statistical tests conducted on transformed daily total means.

The proportions of pullets dust bathing in their rearing pens were similar across rearing treatments (F_(2,6)_ = 1.06, *p* = 0.40), but the proportions decreased from 11 to 14 weeks (F_(1,2)_ = 332.18, *p* = 0.003) with no interaction between treatment and age (F_(2,22)_ = 2.40, *p* = 0.11, Figure 3). There was a significant interaction between age and rearing treatment for the proportion of pullets foraging (F_(2,22)_ = 5.67, *p* = 0.01) with the pullets from the novelty treatment increasing their foraging with age, but the control and structural pullets remained at similar levels between 11 and 14 weeks (Figure 3). Visually, all groups showed similar patterns of dust bathing and foraging across the day (Figure 3).

### 3.2. Free-Range Facility

**Figure 4:**
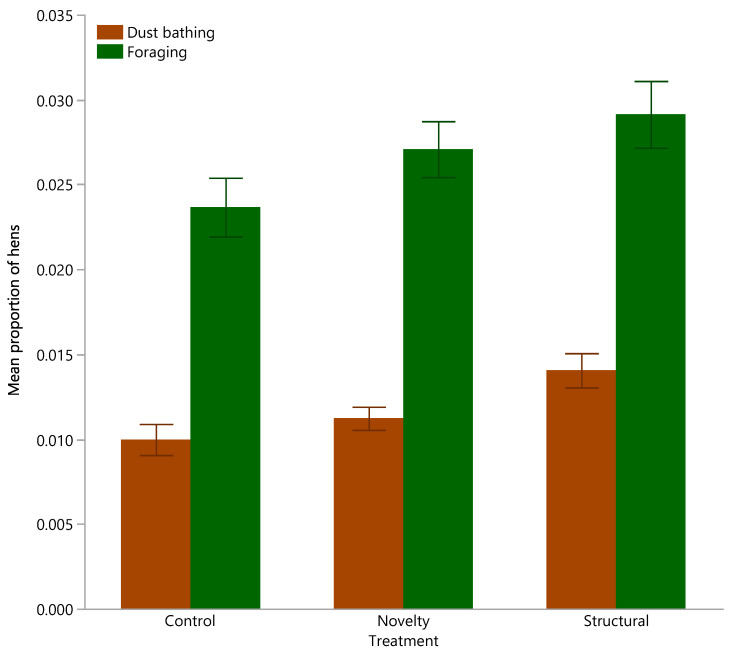
The mean (±SEM) proportion of hens summed for both inside the shed and outside on the range exhibiting dust bathing or foraging behavior across the flock cycle from three rearing treatments (control, novelty, structural). Raw data are presented.

Across all age points there was a significant effect of rearing treatment on the total proportions of adult hens dust bathing (χ^2^ = 13.81, df = 2, *p* = 0.001) and foraging (χ^2^ = 6.53, df = 2, *p* = 0.04) with the structural hens showing more dust bathing and foraging than the control hens only (Figure 4). 

**Figure 5:**
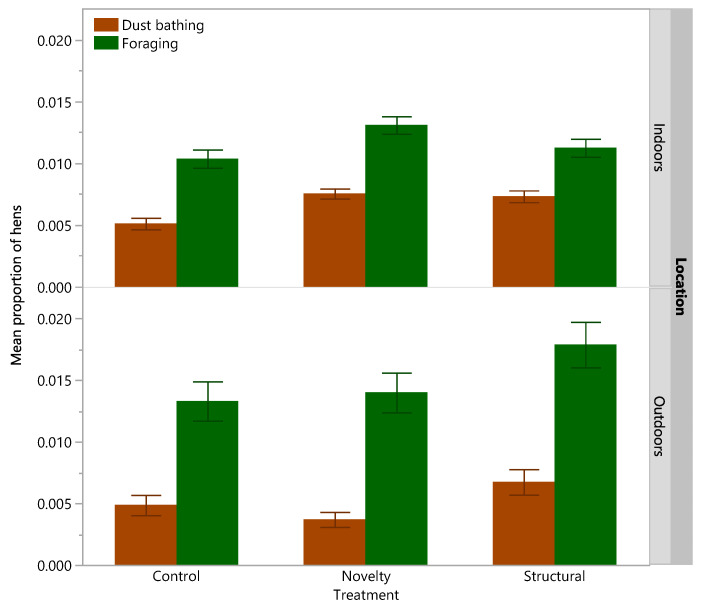
The mean (±SEM) proportion of hens inside the shed (indoors) or outside on the range (outdoors) exhibiting dust bathing or foraging behavior across the flock cycle from three rearing treatments (control, novelty, structural). Raw data are presented.

There was a significant effect of rearing treatment on the proportion of hens dust bathing inside (χ^2^ = 22.54, df = 2, *p* < 0.001) with both the novelty and structural groups showing more dust bathing than the control hens (both *p* ≤ 0.0007, Figure 5). There was a significant effect of rearing treatment on the proportion of hens dust bathing outside (χ^2^ = 11.78, df = 2, *p* < 0.003) with the structural hens showing more dust bathing than the novelty hens only (*p* = 0.002), Figure 5). There was a significant effect of rearing treatment on the proportion of hens foraging inside (χ^2^ = 8.39, df = 2, *p* = 0.02) with the novelty hens showing more foraging than the control hens only (*p* = 0.01, Figure 5). There was also a significant effect of rearing treatment on the proportion of hens foraging outside (χ^2^ = 9.79, df = 2, *p* < 0.008) with the structural hens showing more foraging than the control hens only (*p* = 0.006, Figure 5).

**Figure 6:**
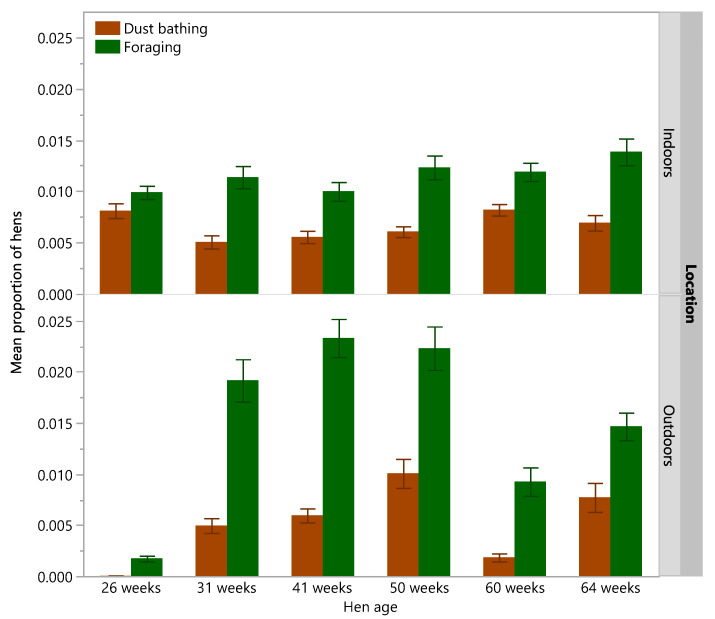
The mean (±SEM) proportion of hens inside the shed (indoors) or outside on the range (outdoors) exhibiting dust bathing or foraging behavior across hen ages (26, 31, 41, 50, 60, 64 weeks). Raw data are presented from all rearing treatments combined.

There were significant differences across age for hens dust bathing inside (χ^2^ = 22.40, df = 5, *p* = 0.0004) and outside (χ^2^ = 64.59, df = 5, *p* < 0.0001) and significant differences across age for hens foraging outside (χ^2^ = 64.55, df = 5, *p* < 0.0001), but not across age for hens foraging inside (χ^2^ = 8.12, df = 5, *p* = 0.15, Figure 6). There was more variation across age for dust bathing and foraging behaviors observed outside on the range than inside the shed (Figure 6).

**Figure 7:**
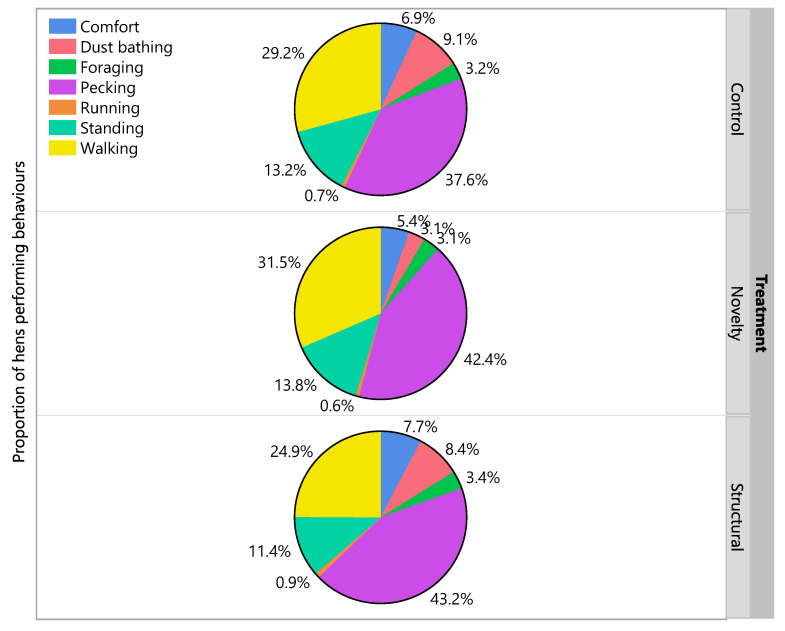
The percentages of hens on a portion of the range performing comfort behaviors, dust bathing, foraging, pecking, running, standing and walking across three different rearing treatments (control, novelty, structural). Observations were made when hens were 50 weeks of age across two days.

Analyses of the time budgets of hens on the range at 50 weeks of age showed no treatment differences in the proportion of hens performing comfort behaviors, dust bathing, foraging, pecking, running, standing and walking (χ^2^ = 0.46–5.10, df = 2, *p* ≥ 0.08; Figure 7). The most frequent behaviors observed were walking, pecking and then standing (Figure 7).

## 4. Discussion

Enrichments provided during the indoor rearing phase for free-range laying hens may have beneficial effects on multiple aspects of behavior and physical health. The aim of this study was to assess how different types of rearing enrichments may affect species-specific, motivated, natural laying-hen behaviors (termed positive behaviors) including foraging and dust bathing for pullets and adult hens across their production cycle as well as play behavior in chicks. Providing novel objects or perching/navigation structures to pullets raised on floor litter had some effects on play behavior but most play behaviors were observed in low frequencies equally among treatments. The novel objects increased the foraging behavior of pullets across age during the rearing treatment phase to a greater degree relative to the control and structural treatment groups. In the adult hens, the perching/navigation structure enrichments increased overall foraging and dust bathing relative to the control hens but there were treatment effects of both enrichment types, dependent on the location and behavior observed. Hens differed in the amount of foraging and dust bathing performed inside the shed versus outside on the range across age. These results demonstrate that for pullets reared on litter, additional enrichments can still result in some increases in these species-specific positive behaviors in the adult hens. These beneficial effects of rearing enrichments may become more apparent in the longer term as the adult birds are exposed to a new environment, come into lay and likely encounter various stressors across the production cycle. 

Rearing enrichments resulted in a greater drop in running behavior relative to the control chicks from 2 to 4 weeks of age but when all play behaviors were combined, there was no effect of the rearing treatments indicating enrichments resulted in minimal impacts on play behaviors. It is possible that a different observation method such as extended continuous observations across fewer periods [32] may have increased observations of play occurrences. All play behaviors did decrease across age which is consistent with other studies [31,32]. The results on enrichment effects are similar to observations of play behavior in broiler chicks provided with enrichment. Broiler results showed no treatment differences in spontaneous play [32] or in play stimulated by personnel walking through and creating an open space [31], but when specific tests of play were conducted, the non-enriched birds were more responsive [32]. The higher proportions of running in the control chicks and greater increase in sparring behavior relative to enriched groups across age may have been a result of few other stimulatory objects in their environment to engage the chicks [32], or the greater open litter space in their comparatively empty pens. Currently the literature on play behavior in chickens is limited and while this study adds knowledge, there is still much to be understood about what stimulates play in young chickens and how this may affect their welfare, both as developing chicks and longer term. 

All birds in this study were raised on litter, which is important for the development of foraging behavior and reductions of feather pecking [21,22], although the effects on dust bathing development are less clear [23,24,25]. The relationship between foraging and feather pecking has been hypothesized to be a redirection of food-related pecking at feathers when a substrate is not present [41], although conflicting results across a multitude of studies highlight the complexities around this relationship [42]. Despite the presence of the litter, there were still some impacts of the rearing enrichments on these behaviors. The perching/navigation structures and novel objects were not intended to specifically increase litter-related behaviors, although there were pecking strings provided as some of the novel objects within some weeks across the 16-week rearing period. These increases suggest that the enrichments had more generalized impacts in optimizing the behavioral development of the birds, resulting in more performance of behaviors that are believed to be positive for laying hens to engage in [43], and that, when thwarted, have been shown to increase the occurrence of abnormal behaviors [11]. 

Increases in foraging only were shown in the novelty pullets across age which may have been a result of changes in the degree of engagement with the varying enrichments in their pens across time. Once hens moved into the laying system and had a choice of engaging in these behaviors both inside the shed in the floor litter, or outside the shed in the dirt, there were differences between rearing treatments for both dust bathing and foraging, but these differences were in part dependent on the location being observed. Overall, the structural hens did show the most foraging and dust bathing, and this in part may have been related to differences in ranging behavior. Through individual range-use tracking using radio-frequency identification technology, the structural hens spent the longest daily time outside on the range, with the novelty and structural hens showing the longest times for individual visits relative to the control birds [34]. This increased time outside with more space may have led to more observations of dust bathing and foraging as previous research with a separate flock in the same experimental setting showed that hens exhibited more of these behaviors outside relative to what was observed inside the shed [12]. However, there were still some treatment effects when comparing just the behavior exhibited inside the shed in the floor litter. It is difficult to conclude from this study whether that was indirectly related to range-use differences among treatment groups (i.e., increased space available inside per hen with more hens outside) or a separate effect of the rearing enrichment that increased the motivation to perform these behaviors. The variation between rearing treatments and behaviors performed inside or outside does highlight how free-range hens have a choice of locations within this type of system and different locations may be preferred for certain behaviors. This can extend to different locations out on the range as well, where open range areas may elicit different behaviors to sheltered areas [14,15]. 

The increase in foraging behavior may have had other welfare benefits across the trial, although confirmation of a causal relationship in this study is limited. The control hens overall showed less foraging relative to the structural hens, and they also exhibited the most plumage damage across time [5]. Foraging is proposed to function as both food searching as well as environmental exploration [11]. In this study, foraging was defined as scratching followed by pecking, but foraging in other studies has encompassed walking, pecking and scratching (e.g., [14]). It is uncertain if the discrepancies in these definitions would also correspond with different motivations behind the behaviors, where walking while pecking may be a greater representation of explorative foraging. In terms of food searching, all adult hens had equal feed available indoors which should have met their nutritional requirements. Thus, it is possible that the structural hens were performing more foraging under increased motivation to explore their environment. This may have resulted from the structures provided during rearing that were intended to improve physical development as well as improve spatial navigation around their pens (each structure included opaque sides designed to provide a visual block for development of navigation abilities) [44]. This would be consistent with the research by Rudkin (2021, [42]), who found no direct correlation between foraging and feather pecking in hens provided with a range of foraging enrichments in their cages, indicating that the foraging substrates enabled development of exploration foraging. If control hens were less engaged in exploring their environments, then this could have increased conspecific pecking behavior and/or stress resulting in this negative pattern of behavior [45]. However, the hens exposed to multiple different novel objects during rearing did not show more foraging overall, only more foraging than the control hens inside the shed. Thus, the relationship between rearing enrichments, exploration, foraging and feather pecking in this study is uncertain and requires further investigation.

## 5. Conclusions

This research demonstrates some long-term benefits of rearing enrichments in the form of novel objects or perching/navigation structures for pullets destined for a free-range environment, where additional complexity in litter-based environments may optimize behavioral development of the pullets and increase performance of positive species-specific behaviors. The effects were most prominent in the pullets reared with the perching/navigation structures throughout development, although the increases were small. Future research should seek to further understand mechanisms behind these effects to design rearing environments that will facilitate desirable behaviors across the laying cycle. Benefits may be seen for laying hens in loose-housed systems with or without range access. 

## Figures and Tables

**Table 1:** Ethogram of the behaviors observed for each hen whilst out on the range at 50 weeks of age.

Behavior	Description
Body shaking	Hen completes a full shake of her body ruffling her feathers
Dust bathing	Hen is lying on the ground, kicking dirt onto her feathers and tossing it over her body with her wings and full body movement
Fighting	Two hens are jumping up and pecking at each other with force
Jumping in air/flying	Hen jumps into air, flaps wings, and travels a short distance
Foraging	Hen scratches her feet backwards in the dirt and then pecks the ground
Pecking	Hen is using her beak to touch the ground or surrounding environment Hen may pick up something (e.g., dirt) with her beak
Pecking other chickens	Hen is using her beak to touch another hen
Piling	Hens are in a group tightly clustered together
Preening	Hen is using her beak on her feathers to align them or pull off debris (e.g., dirt)
Standing	Hen is upright and remaining in one location.
Sunbathing	Hen is lying in the dirt with wings spread out and is motionless (i.e., not moving around as per dust bathing activity)
Running	Hen is upright and moving forward at a fast pace
Tail shaking	Hen shakes tail feathers whilst walking or standing
Walking	Hen is upright and moving forward at a slower pace than when classified as running
Wing flapping	Hen’s wings are outstretched and rapidly flapped while hen remains on the ground (i.e., not airborne)

**Table 2:** The mean (±SEM) percentages of chicks that performed each play behavior (frolicking, wing-flapping and sparring) across three rearing treatments (control, novelty, structural) at three different observation age points (2, 4, 6 weeks). ^a,b^ Dissimilar superscript letters indicate differences across rearing treatments or across age. Raw means are presented with analyses conducted on transformed data.

	Behavior (Mean % ± SEM)	Frolicking	Wing-Flapping	Sparring
Treatment	Control	2.27 ± 0.51	1.12 ± 0.10 ^a^	0.42 ± 0.09
	Novelty	1.75 ± 0.26	1.09 ± 0.07 ^a^	0.29 ± 0.07
	Structural	2.27 ± 0.34	1.0 ± 0.13 ^b^	0.35 ± 0.05
Age	Two weeks	2.43 ± 0.18 ^a^	0.87 ± 0.07 ^b^	0.22 ± 0.04 ^b^
	Four weeks	2.42 ± 0.30 ^a^	1.07 ± 0.06 ^a,b^	0.41 ± 0.06 ^a^
	Six weeks	1.44 ± 0.17 ^b^	1.32 ± 0.10 ^a^	0.43 ± 0.08 ^a,b^

## Data Availability

Data will be made available upon any reasonable request to the corresponding author.
